# Predictors of quitting support from nonsmoking mothers for smoking fathers: a cross-sectional study from Chinese pupils’ families

**DOI:** 10.1186/s12889-024-18217-2

**Published:** 2024-03-05

**Authors:** Nan Jiang, Ling-ling Huo, Zeng-zhi Zhang, Yi-qing Huang, Yu-hua Li, Rui Wang, Yi Guo, Fei Qi, Shan-peng Li

**Affiliations:** 1 Department of Epidemiology and Health Statistics, School of Public Health, Qingdao University, Qingdao, Shandong China; 2 Qingdao West Coast New District Center for Disease Control and Prevention, Qingdao, Shandong China; 3 Qingdao Shinan District Center for Disease Control and Prevention, Qingdao, Shandong China; 4 Qingdao Municipal Center for Disease Control and Prevention, Qingdao, Shandong China; 5 Qingdao Shibei District Center for Disease Control and Prevention, Qingdao, Shandong China; 6 Peking University Clinical Research Institute, Peking University First Hospital, Beijing, China; 7 School of Public Health, Peking University, Beijing, China

**Keywords:** Smoking cessation, Single-smoker couples, Partner support, Family functioning

## Abstract

**Background:**

Quitting support from smokers’ partners can predict quit attempts and smoking abstinence but research on factors that predict such support has been limited. To add more evidence for partner support and the improved interventions for smoking cessation, we analyzed some new potential predictors of quitting support from smokers’ spouses.

**Method:**

This cross-sectional study was conducted in in 2022 and 2023, selecting the students’ families in which fathers smoked and mothers didn’t smoke from grade 1–5 of 13 primary schools in Qingdao, China. Parents who met the criteria completed the online questionnaires and 1018 families were included in the analysis. We measured personal information related to smokers and their spouses such as age, education and nicotine dependence, and variables related to family and marital relationship such as family functioning, perceived responsiveness and power in decision-making of quitting smoking. Quitting support from smokers’ spouses was measured by Partner Interaction Questionnaire and generalized linear model was used to explore the potential predictors of partner support.

**Results:**

In this study, the mean age of smokers was 39.97(SD = 5.57) and the mean age of smokers’ spouses was 38.24(SD = 4.59). The regression analysis showed that for smokers and their spouses, the older age groups showed the lower ratio of positive/negative support(*P* < 0.05) and smokers with high education showed the less positive and negative partner support(*P* < 0.05). Nicotine dependence was positively associated with negative support (*β* = 0.120, *P* < 0.01), and perceived responsiveness (*β* = 0.124, *P* < 0.05) as well as family functioning (*β* = 0.059, *P* < 0.05) was positively associated with positive support. These three factors were associated with ratio of positive/negative support(*P* < 0.05). In addition, power of smoker’s spouse in decision-making of quitting smoking was positively associated with the positive (*β* = 0.087, *P* < 0.001) and negative support (*β* = 0.084, *P* < 0.001).

**Conclusions:**

Nicotine dependence, family functioning, power in decision-making of quitting smoking and perceived responsiveness were found to be the predictors of quitting support from smokers’ spouses. By incorporating predictors of partner support and integrating some established theories that can improve family functioning and marital relationships, smoking cessation interventions can be further improved.

**Supplementary Information:**

The online version contains supplementary material available at 10.1186/s12889-024-18217-2.

## Introduction

As mentioned in World Health Organization(WHO) report on the global tobacco epidemic, reducing tobacco use is critical to reducing the burden of noncommunicable diseases that account for 71% of deaths globally and action for helping tobacco cessation is an essential component of tobacco control strategy [1, 2]. Many countries have established the well-practice clinical guidelines for smoking cessation [3–5] and various smoking cessation interventions have been developed for smokers, such as medication-assisted treatment, psychological behavior therapy or integrated therapy. Although these intervention programs have demonstrated certain effectiveness in smoking cessation [6–8], there are still some challenges such as lack of high levels of efficacy, high relapse rates and low long-term abstinence rates [9–11]. In China, the current smoking cessation services involve short-term smoking cessation interventions in health service settings, smoking cessation consultation hotlines, and smoking cessation clinics [12]. Besides conventional smoking cessation services, the development of mobile technology has brought about novel online smoking cessation service based on the intervention platforms in applications [13–15]. These online and offline smoking cessation services together promote the construction of the smoking cessation service system. However, due to the limited initiative to seek medical care, low quitting motivation and low utilization of smoking cessation services among smokers [16–19], these services cannot fully function at the individual level of smokers. How to further enhance the effectiveness of existing smoking cessation services has been a focus in the field of tobacco control. Supportive factors from the interpersonal network of smokers have received increasing attention. Social support, especially partner support, plays a crucial role in smoking cessation [20–22]. Support from smokers’ partners is generally manifested in various kinds of supportive behaviors, some of which may have a positive influence, such as encouraging the smoker to persevere with quitting or expressing participation, while some kinds are negative, such as criticizing the smoker’s smoking behavior or nagging. The negative and positive quitting support from smoker’s partner are significant predictors of outcomes during different stages of smoking cessation [23–25]. Researchers have endeavored to translate the evidence into efficacious interventions that can optimally tap the role of smoker’s partner in cessation. Previous smoking cessation trials with partner support component used to be conducted in hospitals or health centers and the intervention forms included self-help materials, counselling calls, interview, meeting, etc. These interventions aimed to increase contacts between smokers and their partners and promote supportive behaviors from partners to assist smokers to quit but the results of these trials were mixed [26–29]. A review concluded that interventions failing to promote the partner support led to the limited effects in trials [30] and developing effective interventions targeting smoker’s partner is a research direction that deserves further exploration. One of the trials with significant results in abstinence rate introduced the counseling session, a new form of cessation intervention led by nurses that enhanced the mutual understanding and support between smokers and their spouses through the discussion. It was proved to be effective in promoting partner support but the participation rate was low and efficacy awaits more studies to confirm [28]. More targeted interventions based on heterogeneity of some factors that affect partner support need to be developed, and incorporating improved interventions into various smoking cessation services holds great promise for effectively enhancing partner support and intervention effects. Predictors of partner support will be a key part in the breakthrough of targeted intervention design but the related studies are limited. Michelle et al. revealed that factors such as relationship commitment and satisfaction, concern for health and motivation to quit were associated with partner support [31]. To add more evidence for partner support and establishment of better smoking cessation strategy, we analyzed new potential predictors associated with quitting support from smokers’ spouses.

In recent years, there has been a surge of smoking-related research incorporating family factors and many studies have attempted to develop family-based smoking interventions [32–35]. Cessation interventions combined with family factors emerging as a trend in smoking cessation strategies and the demand for cessation interventions that integrate with family theories is on the rise. Family is a fundamental life unit for human, providing material, psychological and social support for family members’ survival and development, and as a system, the family needs to complete a series of tasks to achieve these basic functions [36]. Family functioning refers to the roles played by family members during various stages of completing household tasks and it can predict mental and physical health, acting as a measure of how well the family system functions [36, 37]. Previous studies revealed that family functioning was associated with some addictive behaviors such as drug and alcohol abuse as well as tobacco use [38–41] with unhealthier family function had a negative effect on the health outcomes. Furthermore, researchers have suggested that the transmission of quitting messages and effects from spouses who received smoking cessation interventions to smokers may be subject to personal factors and family dynamics, leading to potential variations [29]. Therefore, understanding the role and impact of family functioning on quitting support from smokers’ spouses is of great importance to the development of smoking cessation interventions. This study measured family functioning by the instrument based on McMaster model, which is one of the major influential theories in current family assessment theories. This model evaluates family functioning from seven dimensions: problem solving, communication, roles, affective responsiveness, affective involvement, behavior control, and general functioning. Problem solving reflects the family’s ability to effectively address and resolve issues, and the steps in the process. Communication refers to the effectiveness and directness of information exchange within the family. Role describes the efficiency with which family tasks are assigned and completed. Affective responsiveness assesses the family members’ ability to appropriately respond with emotions. Affective involvement pertains to the quality of interest, concern, and investment that family members have for each other. Behavior control refers to the standards and boundaries set for behavior [42]. Since the McMaster model provides a systemic assessment of family functioning and has a broad range of applicability to populations, it has been employed widely in the realm of medicine to explore the relationship between diseases and family functioning by placing individuals within the family system, rather than as separate entities [38–42]. This study aims to explore the impacts behind the family dynamics on quitting support from smokers’ spouses, utilizing the McMaster model as the theoretical framework. To our knowledge, this study is the first to examine the association between family functioning and partner support.

Besides family functioning, perceived responsiveness, a factor reflecting relationship intimacy, was also examined in this study. It reflects the perceptions of the partner’s emotional availability, understanding and responsiveness, and is an important aspect of close relationship [43, 44]. Many studies have suggested that relationship quality is related to the support for partner’s health behavior change, and relationship commitment and satisfaction can predict willingness to provide quitting support for partner [31, 45, 46]. Relationship quality represents the overall health of a relationship, which is measured by various indicators and involves many key elements within the relationship [47]. Responding positively to emotional disclosure can translate into higher relationship satisfaction and commitment [48, 49], and high perceived responsiveness can predict better relationship quality [43]. Yet, the association between perceived responsiveness and partner support has not been examined by previous studies. In addition, studies regarding the smoking cessation have demonstrated that perception of the partner’s support plays a vital role in maintenance of abstinence from smoking [50] and greater perceived responsiveness can predict decreases in smoking over time and better smoking cessation outcomes [44, 51]. We were curious about whether perceived responsiveness of smokers’ spouses was a predictor of partner support and included this factor in this study. Meanwhile, in the assessment of family functioning, the relationships between family members are observed as a whole within the family system. One dimension of the family functioning is affective responsiveness, which reflects the ability to respond to a range of stimuli with appropriate quality and quantity of feelings and it focuses on the pattern of the family’s responses to affective stimuli [37].While perceived responsiveness focuses on perceiving responses from partner in the interactions, acting as a more direct measure of relationship intimacy between spouses and carrying more detailed information about the quality of the marital relationship. Adding family functioning and perceived responsiveness into the study simultaneously could provide more comprehensive evidence for partner support from multiple perspectives.

In addition, upon consulting the prior report regarding the smoking prevalence in Qingdao, we observed that the rates stood at 46.0% for males and 1.1% for females with a much higher prevalence among men than among women [52]. Furthermore, such pattern of notable gender-based disparity exists not only in China but also globally. Report on smoking in China revealed that smoking prevalence of males and females was 50.5% and 2.1%, respectively [53] and WHO global report on trends in prevalence of tobacco use revealed that the proportion of men and women using tobacco was 36.7% and 7.8%, respectively [54]. Additionally, the latest published World Health statistics 2023 pointed out that there was a slower reduction in smoking prevalence among men compared to women with the lack progress among men observed in most WHO regions [55]. Thus, male smokers will be the focal group in tobacco control strategy and this study targeted the male smokers to conduct the survey, furnishing more evidence for this direction of tobacco control.

Given the limited original research as well as the need for improved smoking cessation interventions, this study was aimed at examining predictors of quitting support from smokers’ spouses and providing more clues for promoting the partner support as well as enhancing the intervention effects. This study can cast light on the association between partner support and factors related to personal characteristics, family and marital relationship.

## Methods

### Design and participants

This cross-sectional study was conducted in grades 1–5 of 13 pilot primary schools in Qingdao, China, involving students’ families with smoking fathers and nonsmoking mothers. (Students in sixth grade had graduated prior to the study survey and it was difficult to contact their parents so the sixth grade was not included.) The investigators received standardized training and conducted an initial selection based on the inclusion and exclusion criteria by contacting the students’ parents through phone or face-to-face interview to verify whether they were eligible for the study. The inclusion criteria were: (1) father and mother living with children in the past 30 days; (2) father smoking one or more cigarettes daily in the past 30 days and mother not smoking; (3) familiarity with mobile phone functions. The exclusion criteria were: (1) severe heart, brain, lung, or blood system diseases; (2) a history of mental illness or other medical conditions that prevent completing the questionnaires; (3) father participating in some smoking cessation programs prior to the study survey; (4) parents who were divorced. There were 1251 students’ families that met the criteria in the initial selection. Families with children enrolling in different grades were checked to avoid duplicate records. Then the investigators conducted the further screening in candidate families. Families in which parents declined to participate were excluded. There were 1168 students’ families in the final list of study families and the exclusion proportion was 6.63%. After learning the comprehensive information of study regarding the background, purpose, content of questionnaire, data privacy, and benefits and rights of participation, participants signed the informed consent form in duplicate provided by investigators. These participants were invited to fill out the questionnaires via the online survey platform (“SurveyStar,” Changsha Ranxing Science and Technology, Shanghai, China) in July 2022 and March 2023. The questionnaires were designed into father and mother versions with different measurements. During the data collection period, the investigators urged the parents to complete the questionnaires according to the list of completion status which was regularly fed back from the survey administrators. Questionnaires with missing values can’t be submitted in the platform system. There were 1063 students’ families filling out the questionnaires and the response rate was 84.97%. Every study family was assigned a family number for matching the parental questionnaires. Questionnaires that filled by only one parent in the family, as well as those with abnormal completion time or illogical content were excluded. Finally, we got 1018 valid matched questionnaires with an effective rate of 95.76%.

### Ethical approval

This study has been reviewed and approved by the Institutional Review Board of Qingdao Municipal Center for Disease Control and Prevention, with project number 2021-ZXJK-32.

### Measurement

#### Support for quitting

Support for quitting from nonsmoking mothers to smoking fathers was accessed by Partner Interaction Questionnaire (PIQ), which is most often employed in measuring the partner support and shows adequate reliability and validity [21]. In this study, to ensure the reliability and validity of the Chinese version of PIQ, the translation process was divided into two stages. In the first stage, the Chinese version of PIQ underwent forward and backward translations by the experts in the translation field and was reviewed by the medical and psychology experts to ensure linguistic and cultural equivalence of each item. In the second stage, a pilot survey was conducted before the formal survey and a test of reliability and validity was performed to ensure its applicability in the Chinese context. Previously, this scale was introduced in a smoking cessation trial intervention under an expert system and it also showed good reliability and applicability in the Chinese population [38]. Due to the absence of an officially published Chinese version of PIQ, it was hoped that the work of this study can provide some information for reference to researchers in tobacco control field. Fathers were asked to complete two subscales regarding positive (PIQ-POS) (e.g. “Help you think of substitutes for smoking”, “Express confidence in your ability to quit/remain quit”) and negative support (PIQ-NEG) (e.g. “Criticize your smoking”, “Asked you to quit smoking”) with the instruction “Please answer the following questions about the behaviors of your spouse”. The subscales have 10 items respectively and use a five-point scale from 1(never) to 5(always) with the higher score indicating the higher level of support. In this study, Cronbach’s alpha of positive support and negative subscales were 0.937 and 0.928.The average response of each subscale was the final score and the ratio scores (PIQ-RAT) were created by dividing positive support by negative support [21]. Previous studies have demonstrated that positive and negative behaviors of support are important factors of smoking cessation outcome with PIQ-POS predicting the quit attempts and initial success of abstinence, and PIQ-NEG and PIQ-RAT predicting the continuous abstinence [21, 23, 25, 56, 57]. These three types of PIQ scores will be included in analysis to explore the association between them and potential predictors.

#### Perceived responsiveness

As in the previous studies [44, 58], perceived responsiveness was accessed by the Emotional Intimacy subscale of the Personal Assessment of Intimacy in Relationships (PAIR) [59]. The Chinese version of PAIR showed appropriate reliability and validity [60]. Mothers were asked to complete this scale and the items reflect perceptions of the spouse’s emotional availability (e.g. “I often feel distant from my spouse” [reversed]), understanding (e.g. “My spouse can really understand my hurts and joys”), and responsiveness (e.g. “My spouse listens to me when I need someone to talk to”). There are six items with the response scale from 1 (strongly disagree) to 5 (strongly agree). Responses of six items were averaged to create a final score ranging from 1 to 5 with the higher score indicating the greater perceived responsiveness. In this study, Cronbach’s alpha of this scale was 0.703.

#### Family functioning

Family functioning was accessed by the Family Assessment Device (FAD), a 60-item self-report instrument based on the McMaster model, which contains seven dimensions and shows adequate reliability and internal consistency [42, 61]. The Chinese version of this scale has been tested and shown adequate reliability and validity [61, 62]. The seven subscales are: Problem Solving (PS), Communication (CM), Roles (RL), Affective Responsiveness (AR), Affective Involvement (AI), Behavior Control (BC), and General Functioning (GF). Mothers were asked to respond to each item in terms of “How well it describes your own family.” Each item is rated on a four-point scale from “very much like my home” to “not at all like my home” with the score ranging from 1 to 4. The Cronbach’s alpha of FAD was 0.925. In this study, the responses were averaged to get the mean score of each subscale. According to the healthy/unhealthy cut-off points developed by Miller et al. [63], each dimension of FAD was divided into functional family coded as 1 and dysfunctional family coded as 0. There were seven subscales and the total score ranged from 0 to 7 with the higher score indicating the healthier family functioning.

#### Power in decision-making of quitting smoking

This measurement is self-designed and it reflects the power of smoker’s spouse in the process of determining quitting smoking. The existing researches on decision-making power between spouses mainly focus on daily household decisions, such as consumption and purchasing choices, reproductive decisions [64, 65], and there is no established measurement tool for measuring the power of smoker’s spouse in decision-making regarding smoking cessation. Decision-making interactions between spouses involve the power of involvement, discourse, and decision-making of the subjects and these three types of power have great influence on the outcomes of the decision-making [66]. In this study, three items that could reflect the three types of power in decision-making process to a certain extent were developed. To ensure the validity and applicability of the measurement tool, two measures were taken. Firstly, it underwent the repeated academic discussion and the expert consultation prior to its formal use to ensure that the items were consistent with the intended concepts. Furthermore, a pilot survey was conducted before the formal survey to ensure the reliability and validity of the tool. The three items are: “I often urge my spouse to quit smoking”, “I have a big say in the matter of my spouse quitting smoking” and “My opinion has a great practical impact on the decision for my spouse to quit smoking”, and they are rated on a five-point scale from “strongly disagree” to “strongly agree”. The response options were assigned values from 0 to 4 and the total score ranged from 0 to 12. The higher score indicates the greater influence and power of the smoker’s spouse in decision-making of quitting smoking. In this study, the Kaiser-Meyer-Olkin index of this measurement was 0.724, the Bartlett’s test for sphericity was significant (Chi-Square = 3326.263, *P*<0.001) and Cronbach’s alpha was 0.905. In confirmatory factor analysis, Composite Reliability value was 0.923 and Average Variance Extracted value was 0.801.

#### Individual characteristics

The basic information of students’ parents included age, education, occupation. The age was divided into three groups: 25 ∼ 34, 35 ∼ 44, ≥ 45 years old. Given the possible effects of age difference between parents on quitting support, age difference was calculated as father’s age minus mother’s age. Due to few families in which fathers were more than four years younger than mothers in this study, boundary value of 4 was used in grouping and age difference was divided into five groups:≤-4, -3∼ -1,0, 1 ∼ 3,≥4. Additionally, high school education and below were classified as low education group, coded as 0 and college education and above were classified as the high education group, coded as 1.

#### Nicotine dependence

Nicotine dependence was accessed by the Fagerström-Test for Nicotine Dependence (FTND) [67], which contains six items and establishes the good validity and reliability in Chinese version [68]. Answers of items are scored different points and the total score ranges from 0 to 10 with the higher score indicating the higher level of nicotine dependence. Fathers were asked to complete the items regarding this part.

### Statistical analysis

Statistical analysis was performed using SPSS 24.0(SPSS Inc., Chicago, IL, USA) and R: A language and environment for statistical computing (version 4.2.2, R Foundation for Statistical Computing, Vienna, Austria). *P* values were significant at the level less than 0.05. Descriptive analyses were performed using percentages for categorical variables and means±standard deviations (M±SD) for continuous variables, respectively. Independent samples t-tests and one-way ANOVA or Welch’s Heteroscedastic F Test were used to compare the differences of PIQ scores (PIQ-POS, PIQ-NEG and PIQ-RAT) in categorical variables. Spearman correlation analysis was used to test the correlations between some continuous predictor variables and three types of PIQ scores. Generalized linear model was used to incorporate the variables of individual characteristics, family functioning, perceived responsiveness and power in decision-making of quitting smoking, exploring the association between these factors and PIQ scores.

## Results

### Descriptive statistics and correlation analysis

In 1018 study families, the mean scores of PIQ-POS, PIQ-NEG and PIQ-RAT were 3.66(SD = 1.20), 3.27(SD = 1.19) and 1.24(SD = 0.67). The mean age of fathers was 39.97(SD = 5.57) and the mean age of mothers was 38.24(SD = 4.59). 62.2% of fathers and 64.1% of mothers were high-educated. Statistical analysis showed significant differences in PIQ scores across different groups of age, education and occupation. PIQ-RAT scores showed a significant difference among age groups of fathers and mothers, respectively(*P* < 0.05). High education groups of fathers and mothers showed the lower PIQ-NEG scores and the higher PIQ-RAT scores than the low education groups(*P* < 0.01). Moreover, high education group of fathers had the lower PIQ-POS scores than the low education group(*P* < 0.01). There were significant differences in PIQ-RAT scores across different types of occupations for mothers(*P* < 0.05) and fathers(*P* < 0.01) respectively. Additionally, PIQ-NEG scores showed a significant difference across three types of occupations for fathers(*P* < 0.05). (Table 1)

**Table 1:** Statistical description of personal characteristics of smoking fathers and nonsmoking mothers and PIQ scores

Variables	*N*(M±SD/%)	PIQ-POS (M±SD)	PIQ-NEG (M±SD)	PIQ-RAT (M±SD)
**Father’s age**	1018(39.97±5.57)	3.66±1.20	3.27±1.19	1.24±0.67
25 ∼ 34	137(13.5%)	3.76±1.05	3.21±1.13	1.32±0.71
35 ∼ 44	697(68.5%)	3.62±1.24	3.25±1.22	1.24±0.70
≥ 45	184(18.1%)	3.71±1.14	3.39±1.09	1.16±0.46
***P*** **value**		0.357	0.301	**<0.05**
**Mother’s age**	1018(38.24±4.59)	3.66±1.20	3.27±1.19	1.24±0.67
25 ∼ 34	204(20.0%)	3.74±1.10	3.23±1.21	1.32±0.73
35 ∼ 44	715(70.2%)	3.64±1.23	3.26±1.18	1.23±0.66
≥ 45	99 (9.7%)	3.65±1.17	3.41±1.14	1.14±0.50
***P*** **value**		0.503	0.436	**<0.05**
**Age difference**				
≤-4	26(2.6%)	3.32±1.13	3.01±1.10	1.15±0.44
-3 ∼ -1	159(15.6%)	3.74±1.18	3.31±1.16	1.23±0.59
0	201(19.7%)	3.70±1.23	3.34±1.10	1.23±0.66
1 ∼ 3	424(41.7%)	3.60±1.27	3.24±1.25	1.23±0.70
≥ 4	208(20.4%)	3.73±1.13	3.27±1.19	1.27±0.67
***P*** **value**		0.308	0.668	0.879
**Father’s education**				
Low education	385(37.8%)	3.78±1.11	3.53±1.13	1.16±0.57
High education	633(62.2%)	3.59±1.25	3.12±1.19	1.28±0.71
***P*** **value**		**<0.01**	**<0.001**	**<0.01**
**Mother’s education**				
Low education	365(35.9%)	3.72±1.16	3.48±1.13	1.14±0.52
High education	653(64.1%)	3.63±1.22	3.16±1.20	1.29±0.73
***P*** **value**		0.262	**<0.001**	**<0.001**
**Mother’s occupation**				
White-collar	281(27.6%)	3.64±1.21	3.16±1.20	1.30±0.75
Blue-collar	58(5.7%)	3.35±1.24	3.19±1.15	1.09±0.35
Other	679(66.7%)	3.70±1.19	3.33±1.18	1.23±0.64
***P*** **value**		0.095	0.111	**<0.05**
**Father’s occupation**				
White-collar	312(30.6%)	3.68±1.15	3.23±1.19	1.29±0.76
Blue-collar	155(15.2%)	3.62±1.26	3.50±1.21	1.09±0.40
Other	551(54.1%)	3.66±1.21	3.23±1.17	1.25±0.66
***P*** **value**		0.881	**<0.05**	**<0.01**
**Father’s nicotine dependence**	1018(2.08±2.21)	3.66±1.20	3.27±1.19	1.24±0.67

Based on the questionnaire regarding family and marital relationship, the results showed that the mean score of perceived responsiveness, power in decision-making of quitting smoking and family functioning was 3.85(SD = 0.83), 7.93(SD = 3.39) and 4.35(SD = 2.13), respectively. The study found the significant differences in PIQ-scores between groups in different dimensions of family functioning. PIQ-POS scores varied between functional and dysfunctional groups in six dimensions of family functioning, including CM, RL, AR, AI, BC, GF with functional groups having the higher scores(*P* < 0.01). PIQ-RAT scores varied between functional and dysfunctional groups in all dimensions of family functioning with functional groups having the higher scores(*P* < 0.01). (Table 2)

**Table 2:** Statistical description of perceived responsiveness, power in decision-making of quitting smoking, family functioning and PIQ scores

Variables	*N*(M±SD/%)	PIQ-POS (M±SD)	PIQ-NEG (M±SD)	PIQ-RAT (M±SD)
**Perceived responsiveness**	1018(3.85±0.83)	3.66±1.20	3.27±1.19	1.24±0.67
**Power in decision-making of quitting smoking**	1018(7.93±3.39)	3.66±1.20	3.27±1.19	1.24±0.67
**Family functioning**	1018(4.35±2.13)	3.66±1.20	3.27±1.19	1.24±0.67
**PS dimension**				
PS-functional	776(76.2%)	3.69±1.18	3.24±1.17	1.27±0.68
PS-dysfunctional	242(23.8%)	3.56±1.26	3.39±1.23	1.14±0.60
***P*** **value**		0.134	0.080	**<0.01**
**CM dimension**				
CM-functional	724(71.1%)	3.73±1.19	3.24±1.17	1.28±0.68
CM-dysfunctional	294(28.9%)	3.49±1.21	3.35±1.22	1.15±0.62
***P*** **value**		**<0.01**	0.159	**<0.01**
**RL dimension**				
RL-functional	800(78.6%)	3.73±1.20	3.25±1.20	1.28±0.70
RL-dysfunctional	218(21.4%)	3.39±1.18	3.33±1.15	1.09±0.49
***P*** **value**		**<0.001**	0.418	**<0.001**
**AR dimension**				
AR-functional	642(63.1%)	3.80±1.18	3.28±1.20	1.30±0.72
AR-dysfunctional	376(36.9%)	3.43±1.20	3.26±1.60	1.14±0.55
***P*** **value**		**<0.001**	0.819	**<0.001**
**AI dimension**				
AI-functional	663(65.1%)	3.75±1.21	3.25±1.21	1.30±0.74
AI-dysfunctional	355(34.9%)	3.50±1.17	3.31±1.14	1.13±0.48
***P*** **value**		**0.001**	0.479	**<0.001**
**BC dimension**				
BC-functional	238(23.4%)	4.03±1.16	3.24±1.25	1.43±0.81
BC-dysfunctional	780(76.6%)	3.55±1,19	3.28±1.17	1.18±0.60
***P*** **value**		**<0.001**	0.615	**<0.001**
**GF dimension**				
GF-functional	591(58.1%)	3.82±1.17	3.23±1.18	1.32±0.72
GF-dysfunctional	427(41.9%)	3.44±1.20	3.32±1.19	1.12±0.56
***P*** **value**		**<0.001**	0.240	**<0.001**

The correlation analysis provided a preliminary exploration of the relationship between continuous predictor variables and three types of PIQ scores. The results showed that perceived responsiveness (*r*_*s*_=0.197), family functioning (*r*_*s*_=0.193) and power in decision-making of quitting smoking (*r*_*s*_=0.272) were positively correlated with PIQ-POS scores (*P* < 0.001) while nicotine dependence was negatively correlated with PIQ-POS scores (*r*_*s*_=-0.074, *P* < 0.05). Both nicotine dependence (*r*_*s*_=0.069, *P* < 0.05) and power in decision-making of quitting smoking (*r*_*s*_=0.200, *P* < 0.001) were positively correlated with PIQ-NEG scores. Perceived responsiveness (*r*_*s*_=0.200) and family functioning (*r*_*s*_=0.221) were positively correlated with PIQ-RAT scores and nicotine dependence (*r*_*s*_=-0.137) was negatively correlated with PIQ-RAT scores(*P* < 0.001). (Table 3)

**Table 3:** Correlations between predictor variables and three types of PIQ scores

Variables	PIQ-POS	PIQ-NEG	PIQ-RAT
Nicotine dependence	**-0.074** ^*^	**0.069** ^*^	**-0.137** ^***^
Perceived responsiveness	**0.197** ^***^	-0.023	**0.200** ^***^
Family functioning	**0.193** ^***^	-0.049	**0.221** ^***^
Power in decision-making of quitting smoking	**0.272** ^***^	**0.200** ^***^	0.058

^***^P < 0.001, ^*^P < 0.05

### Regression analysis

The results of the generalized linear model revealed the factors associated with PIQ scores. Given the age difference was calculated by parental age, there was a high correlation between parents’ age and age difference. Two models were established to analyze the association between parents’ age and PIQ scores separately. Model 1 and 2 included the variable of father’s age and mother’s age respectively, and age difference, other explanatory and control variables were incorporated within both of the two models. The results of father’s age in Model 1 as well as the complete results of Model 2 are shown in Table 4. The results of Model 1 were similar to those of Model 2 (see Supplementary Table 1). The analysis of fathers’ age in Model 1 revealed that compared to the age group 25 to 34, the age group 45 and above showed the lower PIQ-RAT scores (*β*=-0.189, *P* < 0.05). The similar result was also found in age groups of mothers in Model 2. Compared to the mothers aged 25 to 34 years, mothers aged 35 to 44 years (*β*=-0.141, *P* < 0.01) and 45 years and above (*β*=-0.160, *P* < 0.05) showed the lower PIQ-RAT scores. Moreover, compared to the fathers with low education, fathers with high education showed the lower scores of PIQ-POS (*β*=-0.236, *P* < 0.05) and PIQ-NEG (*β*=-0.287, *P* < 0.01). Nicotine dependence of smoking fathers was positively associated with PIQ-NEG scores (*β* = 0.120, *P* < 0.01) and negatively associated with PIQ-RAT scores (*β*=-0.072, *P* < 0.01). Additionally, variables related to family and marital relationship had associations with PIQ scores. Family functioning was positively associated with PIQ-POS scores (*β* = 0.059, *P* < 0.05) and PIQ-RAT scores (*β* = 0.035, *P* < 0.05). Perceived responsiveness was positively associated with PIQ-POS scores (*β* = 0.124, *P* < 0.05) and PIQ-RAT scores (*β* = 0.087, *P* < 0.01). It’s worth noting that both of these two factors were not related to PIQ-NEG. Power of nonsmoking mothers in decision-making of quitting smoking was positively associated with scores of PIQ-POS (*β* = 0.087, *P* < 0.001) and PIQ-NEG (*β* = 0.084, *P* < 0.001). (Table 4)

**Table 4:** Estimates of factors associated with PIQ scores in generalized linear model

Explanatory variables	PIQ-POS		PIQ-NEG		PIQ-RAT	
	*β*	*P* value	*β*	*P* value	*β*	*P* value
**Model 1**						
**Father’s age**						
25 ∼ 34	Reference		Reference		Reference	
35 ∼ 44	-0.083	0.459	0.127	0.253	-0.120	0.057
≥ 45	-0.102	0.472	0.129	0.361	**-0.189** ^*^	**0.018**
**Model 2**						
**Mother’s age**						
25 ∼ 34	Reference		Reference		Reference	
35 ∼ 44	-0.073	0.430	0.148	0.110	**-0.141** ^**^	**0.007**
≥ 45	-0.116	0.418	0.126	0.376	**-0.160** ^*^	**0.047**
**Age difference**						
0	Reference		Reference		Reference	
≤-4	-0.238	0.321	-0.390	0.103	0.041	0.762
-3 ∼ -1	0.032	0.791	-0.055	0.647	0.021	0.759
1 ∼ 3	-0.144	0.142	-0.063	0.521	-0.030	0.584
≥ 4	-0.018	0.874	-0.106	0.349	0.048	0.448
**Father’s education**						
Low education	Reference		Reference		Reference	
High education	**-0.236** ^*^	**0.013**	**-0.287** ^**^	**0.002**	0.001	0.993
**Mother’s education**						
Low education	Reference		Reference		Reference	
High education	-0.003	0.976	-0.105	0.283	0.084	0.128
**Father’s occupation**						
Blue-collar	Reference		Reference		Reference	
White-collar	0.091	0.467	-0.142	0.253	**0.150** ^*^	**0.033**
Other	0.058	0.589	**-0.228** ^*^	**0.033**	**0.147** ^*^	**0.015**
**Mother’s occupation**						
Blue-collar	Reference		Reference		Reference	
White-collar	0.334	0.059	0.179	0.310	0.120	0.231
Other	**0.357** ^*^	**0.027**	0.240	0.135	0.092	0.312
**Nicotine dependence**	-0.004	0.923	**0.120** ^**^	**0.001**	**-0.072** ^**^	**0.001**
**Family functioning**	**0.059** ^*^	**0.023**	-0.030	0.243	**0.035** ^*^	**0.016**
**Perceived responsiveness**	**0.124** ^*^	**0.021**	-0.016	0.759	**0.087** ^**^	**0.004**
**Power in decision-making of quitting smoking**	**0.087** ^***^	**<0.001**	**0.084** ^***^	**<0.001**	-0.011	0.088

^***^P < 0.001, ^**^P < 0.01, ^*^P < 0.05

## Discussion

This study gave insights into the factors associated with quitting support from smokers’ spouses and added novel findings for tobacco control. The smokers and their spouses in this study were from families of primary school students in Qingdao, mainly in the young and middle age groups. The multivariable analysis demonstrated the different predictive abilities of the factors related to personal characteristics, family and marital relationship for quitting support from spouses.

Based on the analysis of personal characteristics of smokers and their spouses, we found that age, education levels of smokers and nicotine dependence were associated with partner support. The results of age groups from two models showed that for smokers and their spouses, compared to the younger age group, the older age group had the lower ratio of positive/negative support. Different age groups represent different life stages and different marriage duration. The changes in ratio of two support indicated that changes in interaction patterns and marital relationships with transitions of life stages might affect some types of supportive behaviors and larger population sample is needed to examine the associations between age-related factors and partner support in the future. Additionally, the results also indicated that smokers with high education had less positive and negative support than counterparts with low education but the ratio of two support showed no difference between education level groups. Previous literatures have found that the higher education level is associated with higher willingness to quit smoking and more quit attempts [69, 70], and such initiative in behavior changes may reduce the need of partner support. Moreover, the results showed that among smokers, greater nicotine dependence was associated with more negative support and the lower ratio of positive/negative support and it didn’t demonstrate predictive ability for positive support. Previous studies have indicated that smokers with greater nicotine dependence have lower intentions for behavior change and lower smoking abstinence self-efficacy [71, 72]. Severe nicotine dependence is a risk factor for success of smoking cessation [73], and higher tobacco cravings as well as multiple relapses may trigger more complaints or other negative behaviors regarding quitting smoking from spouses. Targeted interventions can be developed for spouses of smokers with different levels of nicotine dependence to enhance the effectiveness of spousal interventions.

This study provided novel and important information that perceived responsiveness, power in decision-making of quitting smoking and family functioning were associated with quitting support from smokers’ spouses. The evidence showed that perceived responsiveness of smoker’s spouse did affect the partner support with higher perceived responsiveness associated with higher positive support and higher ratio of positive/negative support. Previous studies focused upon impact of perceived responsiveness of smokers and found that smokers who reported greater perceived responsiveness had a decrease in cigarette quantity over years [44, 51]. For smokers, general perceptions of the partner’s availability and willingness to provide support are important for reducing smoking [44] and such perception also counts for smokers’ spouses. A study revealed that perceived spouse responsiveness can predict the own responsiveness to the spouse [74], so in high perceived responsiveness, there is a positive bidirectional influence between smoker’s quitting behavior and spouse’s supportive behavior, which may promote the maintenance of effect of partner support in long-term smoking cessation and improving health outcomes. Future smoking cessation strategy can take marital relationship quality into consideration and develop interventions that enhance effects of partner support by improving the perceived responsiveness of couples. Moreover, the findings in this study cast light on the association between partner support and power of smoker’s spouse in decision-making of quitting smoking. The results revealed that families in which smokers’ spouses had more power in decision-making of quitting smoking showed more positive and negative quitting support. The power of involvement, discourse, and decision-making were involved in decision-making interactions and the distribution of power in decision-making can reflect the interaction mode and power perception between spouses [66]. The decision-making of quitting smoking led by smoker’s spouse can predict the high frequency of supportive behaviors. The targeted interventions should be developed for smokers’ spouses with less power in decision-making of quitting smoking to promote their engagement in quitting smoking and enhance the involvement power. It’s worth noting that power of smoker’s spouse in decision-making of quitting smoking had no association with PIQ-RAT in this study. PIQ-RAT has been proved to be a more important predictor of abstinence in longer cessation maintenance than positive or negative support alone [21]. Although spouses who have great power and influence in decision-making of quitting smoking showed more support for quitting smoking, this pattern of decision-making with an imbalance of power might have limited impact on long-term smoking cessation.

This study successfully detected that the healthier family functioning was associated with more positive support and higher ratio of positive/negative support. Family functioning has been proved to be related to partner daily communication [75], and partner support, as a measure of interaction and communication between spouses in terms of quitting smoking, is influenced by the family system. The healthier family functioning reflects the better interaction and affective involvement among family members, and spouses may show more concerns and provide more support for smokers in well-functioning families. Meanwhile, the better behavioral control pattern, which involves a reasonable standard and reasonable amount of flexibility, tends to be established in families with good functioning [37]. Effective behavior management reflects the flexible and acceptable rules and good health behavior promotion mechanisms, which implies that in families with healthier functioning, spouses are more likely to provide positive feedback and support towards quitting smoking. Furthermore, evidence in this study showed that family functioning had no association with negative support. A study indicated that people with good intentions of changing their partners’ smoking behavior may translate those intentions into potentially counterproductive influence strategies [31] and we inferred that, despite greater involvement from spouses in smokers in healthier family systems, some of the involvement may turn into ineffective support or negative emotions and behaviors. This could potentially attenuate the effect of family functioning on negative support behaviors. More research is needed to validate these findings in the future and delve deeper into the reasons for the relationship between family functioning and partner support. Based on the findings in this part, there are some implications for the future family-based interventions of smoking cessation that it should tailor the specific smoking cessation interventions for both the smokers and their spouses according to the characteristics of families with different levels of functioning. Additionally, the predictive ability of family functioning on different forms of partner support suggested that the improvement of family functioning could be beneficial to smokers in the matter of quitting smoking and family theories can be integrated into interventions of smoking cessation to achieve the better cessation outcomes. Previous study successfully detected the significant positive effects of family therapy on family functioning among couples based on McMaster model [76] and the application of such family therapy in smoking cessation will be a promising thing, as it has the potential to enhance partner support and the effects of interventions by improving family functioning.

Factors examined in this study demonstrated the different predictive ability for different forms of support. Among them, age, nicotine dependence, family functioning and perceived responsiveness demonstrated the predictive ability for ratio of positive/negative support, which is an important predictor of abstinence in longer cessation maintenance. The study also carried valuable and new evidence to the smoking cessation strategy. Interventions for smokers’ spouses should take account of the heterogeneity within different predictors, such as different levels of nicotine dependence to develop different interventions for the targeted groups. What’s more, smoking cessation interventions can be developed in conjunction with family-related and marital relationship factors, by integrating smoking cessation interventions with other established intervention theories which could improve family functioning and spousal relationship quality. This may improve the effectiveness of interventions to some extent and facilitate the establishment of smoke-free environments in households, contributing to the improved health outcomes.

There also existed some limitations in this study. Firstly, data was from pilot schools in one city of China and the age range of participants didn’t cover all age groups, which may lead to insufficient representativeness of the sample. Given the limited nature of the sample, it is possible that the results do not generalize to other populations. Secondly, some variables such as quitting support, family functioning and perceived responsiveness were provided by smokers or smokers’ spouses and it awaits future studies to collect data on these variables from both smokers and their spouses, comparing them to identify similarities and differences in the results of couples. Thirdly, we only studied one type of partner(spouses) and the conclusions of this study cannot be applied to other types of partners, such as people in romantic relationship or close friends who can also provide partner with support. Additionally, this study focused on the quitting support from nonsmoking female spouses to male smokers, smoking status and sex of smokers’ spouses might have influence on results and this needs more studies to examine in the future. Lastly, all the data were self-reported and the quality of the answer may also lead to bias in the survey.

## Conclusions

This study finds some predictors of quitting support from smokers’ spouses, which are related to the personal characteristics, family and marital relationship. The evidence reveals that for smokers and their spouses, the older age groups show the lower ratio of positive/negative support. Smokers with high education tend to have less positive and negative partner support and nicotine dependence positively predicts negative partner support. Furthermore, the healthier family functioning and the higher perceived responsiveness can predict more positive partner support and higher ratio of positive/negative support. Power of smoker’s spouse in decision-making of quitting smoking positively predicts the positive and negative partner support. The study highlights the importance of incorporating novel factors such as power in decision-making of quitting smoking, family functioning and perceived responsiveness into the smoking cessation interventions. What’s more, the results cast light on a possible direction for smoking cessation strategies that interventions can be integrated with some established theories that can improve family functioning and marital relationship. The evidence from this study has implications for the improved smoking cessation strategies and provides more clues to overcome the bottleneck in interventions with limited effectiveness for smokers’ spouses.

### Electronic supplementary material

Below is the link to the electronic supplementary material.

Supplementary Material 1

## Data Availability

The datasets generated and/or analyzed during the current study are not publicly available due to the guarantee of the confidentiality of personal and health information but are available from the corresponding author on reasonable request.

## Abbreviations

WHO: World Health Organization
RCTs: Randomized controlled trials
PIQ: Partner Interaction Questionnaire
PIQ-POS: Positive support in Partner Interaction Questionnaire
PIQ-NEG: Negative support in Partner Interaction Questionnaire
PIQ-RAT: Ratio of positive/negative support
PAIR: Personal Assessment of Intimacy in Relationships
FAD: Family Assessment Device
PS: Problem Solving
CM: Communication
RL: Roles
AR: Affective Responsiveness
AI: Affective Involvement
BC: Behavior Control
GF: General Functioning
FTND: Fagerström-Test for Nicotine Dependence
M: Mean
SD: Standard deviation
