# Supplementary material for: Predictors of quitting support from nonsmoking mothers for smoking fathers: a cross-sectional study from Chinese pupils’ families
Source: BMC Public Health. 2024 Mar 5;24:709. doi: 10.1186/s12889-024-18217-2 (PMC10916209; doi:10.1186/s12889-024-18217-2)
Supplement: Supplementary file 1 — Supplementary Material 1 [file 12889_2024_18217_MOESM1_ESM.docx]

**Supplementary Table 1** Estimates of factors associated with PIQ scores in generalized linear model (Model 1)

| **Explanatory variables** | **PIQ-POS** | |  | **PIQ-NEG** | |  | **PIQ-RAT** | |
| --- | --- | --- | --- | --- | --- | --- | --- | --- |
|  | ***β*** | ***P* value** |  | ***β*** | ***P* value** |  | ***β*** | ***P* value** |
| **Father’s age** |  |  |  |  |  |  |  |  |
| 25~34 | Reference |  |  | Reference |  |  | Reference |  |
| 35~44 | -0.083 | 0.459 |  | 0.127 | 0.253 |  | -0.120 | 0.057 |
| ≥45 | -0.102 | 0.472 |  | 0.129 | 0.361 |  | **-0.189^*^** | **0.018** |
| **Age difference** |  |  |  |  |  |  |  |  |
| 0 | Reference |  |  | Reference |  |  | Reference |  |
| ≤-4 | -0.293 | 0.224 |  | -0.318 | 0.187 |  | -0.039 | 0.776 |
| -3 ~ -1 | 0.017 | 0.886 |  | -0.035 | 0.771 |  | -0.001 | 0.992 |
| 1 ~ 3 | -0.132 | 0.179 |  | -0.084 | 0.393 |  | -0.006 | 0.909 |
| ≥4 | 0.005 | 0.966 |  | -0.134 | 0.265 |  | 0.101 | 0.137 |
| **Father’s education** |  |  |  |  |  |  |  |  |
| Low education | Reference |  |  | Reference |  |  | Reference |  |
| High education | **-0.235^*^** | **0.014** |  | **-0.290^**^** | **0.002** |  | 0.001 | 0.996 |
| **Mother’s education** |  |  |  |  |  |  |  |  |
| Low education | Reference |  |  | Reference |  |  | Reference |  |
| High education | -0.001 | 0.989 |  | -0.098 | 0.316 |  | 0.073 | 0.183 |
| **Father’s occupation** |  |  |  |  |  |  |  |  |
| Blue-collar | Reference |  |  | Reference |  |  | Reference |  |
| White-collar | 0.083 | 0.506 |  | -0.131 | 0.291 |  | **0.140^*^** | **0.046** |
| Other | 0.055 | 0.609 |  | **-0.224^*^** | **0.037** |  | **0.142^*^** | **0.020** |
| **Mother’s occupation** |  |  |  |  |  |  |  |  |
| Blue-collar | Reference |  |  | Reference |  |  | Reference |  |
| White-collar | 0.335 | 0.059 |  | 0.187 | 0.290 |  | 0.119 | 0.236 |
| Other | **0.359^*^** | **0.026** |  | 0.246 | 0.126 |  | 0.093 | 0.306 |
| **Nicotine dependence** | -0.001 | 0.970 |  | **0.115^**^** | **0.002** |  | **-0.067^**^** | **0.002** |
| **Family functioning** | **0.058^*^** | **0.026** |  | -0.029 | 0.258 |  | **0.034^*^** | **0.018** |
| **Perceived responsiveness** | **0.125^*^** | **0.020** |  | -0.017 | 0.744 |  | **0.086^**^** | **0.005** |
| **Power in decision-making of quitting smoking** | **0.087^**^** | **<0.001** |  | **0.083^**^** | **<0.001** |  | -0.011 | 0.115 |

^***^*P*<0.001, ^**^*P*<0.01, ^*^*P*<0.05
